# Bile Duct Tumor as the Presenting Manifestation of Colon Cancer: A Case Report

**DOI:** 10.7759/cureus.46378

**Published:** 2023-10-02

**Authors:** Krystal Mills, Allan Joseph, Adedayo Abioye, Phuong Nguyen, Jabez Beazer, Chima Amadi, Muhammad Bilal, Pramod Pantangi

**Affiliations:** 1 Gastroenterology and Hepatology, Mayo Clinic, Rochester, USA; 2 Internal Medicine, Morehouse School of Medicine, Atlanta, USA; 3 Internal Medicine, Ascension St. Vincent’s East Hospital, Birmingham, USA; 4 Gastroenterology, Marshall University Joan C. Edwards School of Medicine, Huntington, USA

**Keywords:** colon cancer survillence, bile duct cancer, painless obstructive jaundice, bile duct diseases, cancer colon

## Abstract

Painless obstructive jaundice is a well-recognized clinical sign of hepatocellular pathology or obstruction of the biliary system. Bile duct tumors are a known etiology of painless obstructive jaundice. Here, we present a case of obstructive jaundice, which was initially thought be caused by cholangiocarcinoma based on computerized tomography imaging and endoscopic retrograde cholangiopancreatography but was later found to be hilar metastasis from an undiscovered colon cancer.

## Introduction

Bile duct tumors often arise de novo from the proliferation of epithelial cells within the common bile duct and may be further classified by their anatomical location or malignant potential [1-5]. Although colorectal cancer (CRC) can metastasize to the liver parenchyma, metastasis to the common bile duct (CBD) as an etiology of obstructive jaundice is exceedingly rare [5-11]. In the existing literature, these have been found in patients with a pre-existing diagnosis of colon cancer [8-11]. To our knowledge, this is the first reported case of a bile duct tumor leading to the diagnosis of metastatic colon cancer.

This case report was previously presented as a meeting abstract at the American College of Gastroenterology Annual Scientific Meeting in October 2020.

## Case presentation

A 61-year-old man with no known medical history presented with nausea, vomiting, jaundice, and dark urine for two weeks. Review of systems was otherwise negative. He was a tobacco user and had four tattoos. He denied alcohol, intravenous, or other drug use. He had no recent use of acetaminophen or herbal medications. He had no recent travel, exposure to ill contacts, history of hepatitis, or risky sexual practices. He had no known personal history of inflammatory bowel disease or gastrointestinal malignancy.

**Figure 1:**
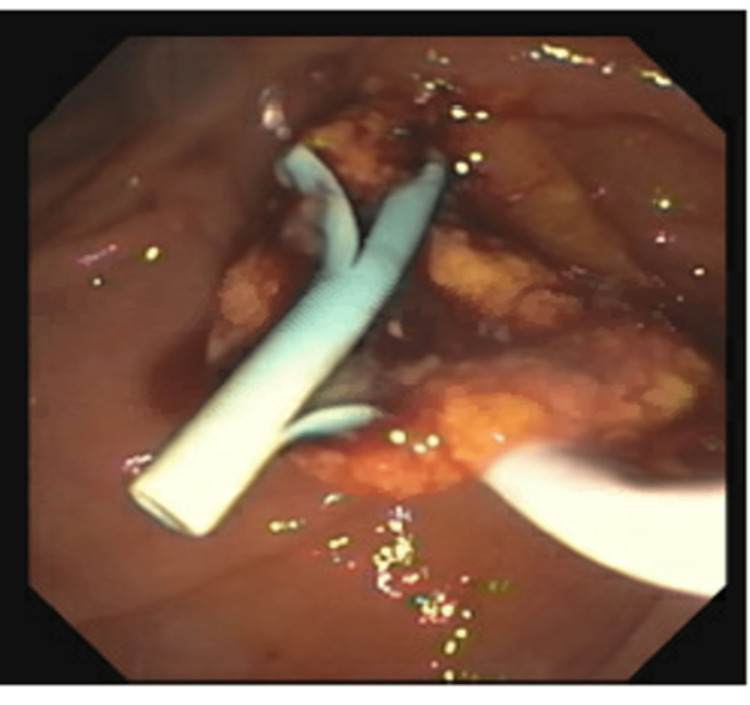
ERCP image showing tumor extruding from the CBD ERCP: endoscopic retrograde cholangiopancreatography; CBD: common bile duct

His vitals were within normal limits on presentation. On general examination, the patient was an elderly male with scleral icterus, but other systems, including the abdomen, were unremarkable. CT abdomen revealed a porta hepatis mass at the CBD, concerning for cholangiocarcinoma. There were also multiple focal areas of the liver, involving the right lobe extending into segment 4, worrisome for additional large areas of metastatic cholangiocarcinoma. He was admitted for further workup. Endoscopic ultrasound was performed and endoscopic retrograde cholangiopancreatography (ERCP) revealed a tumor extruding from the CBD (Figure 1).

**Figure 2:**
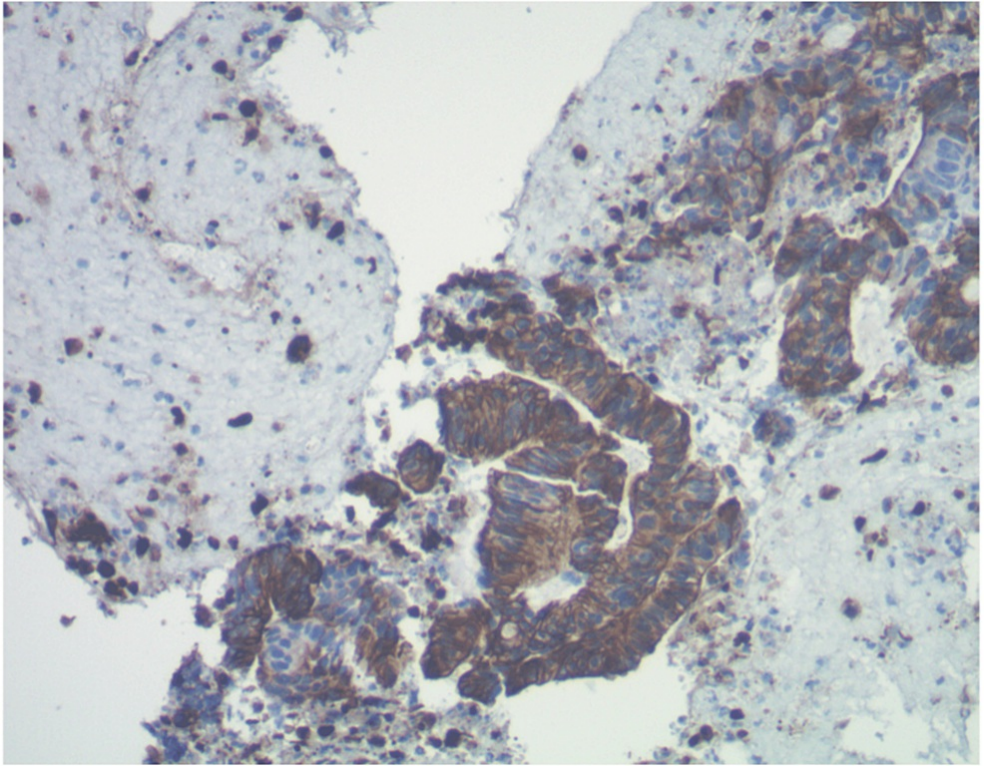
Immunohistochemical stain for common bile duct biopsy, showing CK20 positivity

The CBD mass and a hepatic mass were biopsied, and a stent was placed. Immunohistochemical staining of the biopsies were negative for CK7 but positive for CK20 and CDX2, in keeping with a colonic primary, but there was no clinical evidence of a primary CRC (Figure 2).

**Figure 3:**
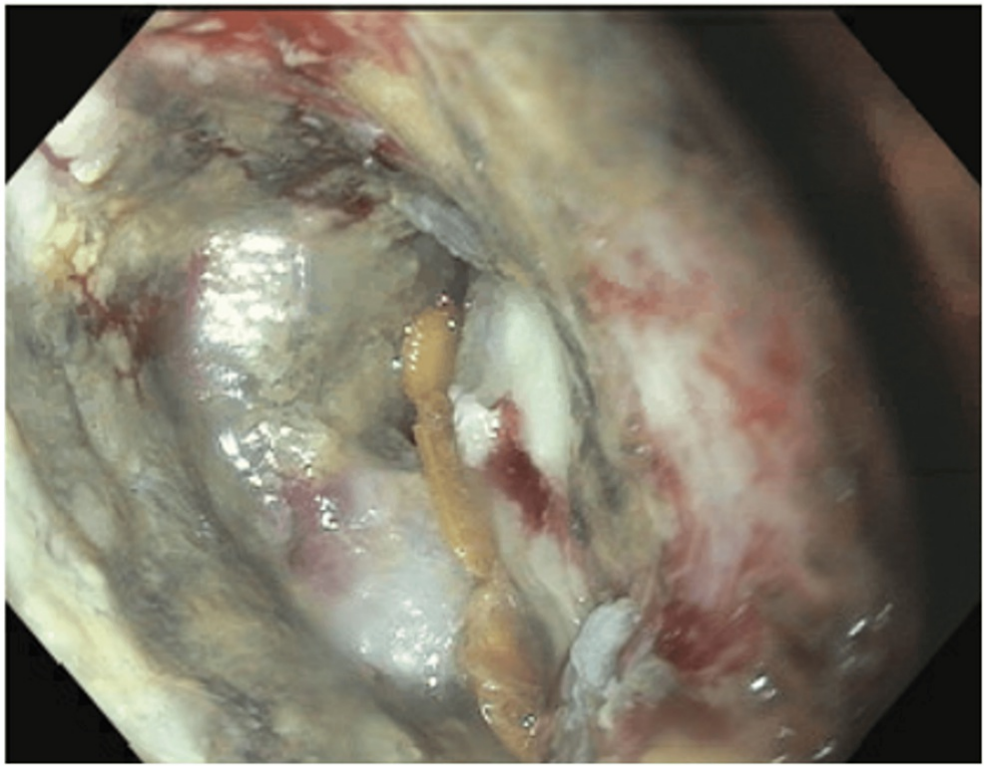
Colonoscopy image revealing mass in the sigmoid colon

**Figure 4:**
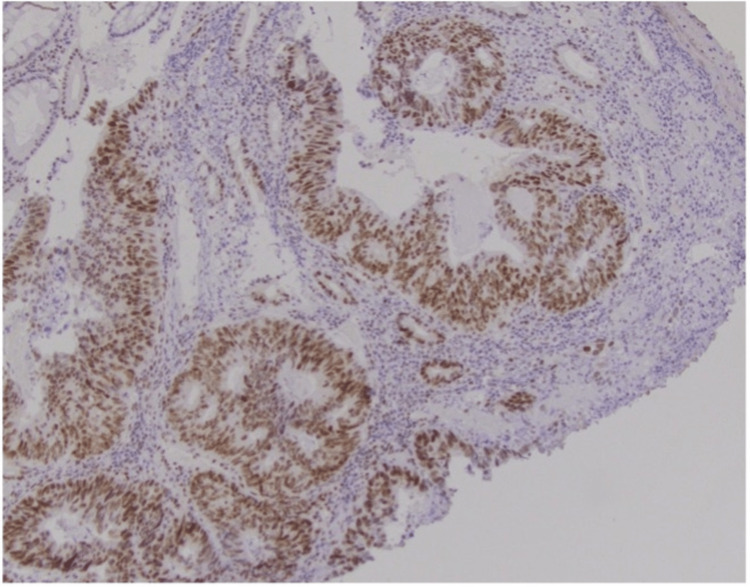
Immunohistochemical stain for MLH1, one of the four stains in the mismatch repair (MMR) panel (the other three stains were also positive)

In exceedingly rare cases, a primary cholangiocarcinoma can exhibit intestinal differentiation. For the completion of evaluation, a subsequent colonoscopy was performed. Colonoscopy revealed an ulcerative, partially obstructive mass in the sigmoid colon with histology showing adenocarcinoma with four stains in the mismatch repair (MMR) panel positive (Figures 3, 4).

The patient was scheduled for oncology follow-up.

## Discussion

Bile duct neoplasms may arise from the proliferation of cholangiocytes along the intrahepatic or extrahepatic portions of the bile duct [1-5]. They may be further categorized as benign or biliary tumor precursors. Benign tumors include bile duct adenomas, biliary micro-hamartomas, biliary adenofibromas, and mucinous cystic neoplasms of the liver and biliary system. Biliary tumor precursors include biliary intraepithelial neoplasia and intraductal papillary neoplasm of the bile ducts [12]. These tumors often arise de novo in the biliary tree.

There have been exceedingly rare cases of patients with a history of colon cancer developing metastasis to the common bile duct, including after the treatment of their primary malignancy [8-11]. A retrospective study estimated that, in metastatic CRC, major bile duct involvement occurs in 1.9% of cases and minor bile duct occurs in 1.7% of cases [8]. The clinical features may vary based on the extent of intrabiliary growth. Major duct involvement is more likely to display obstructive liver chemistries, radiographic evidence of disease, and sclerosing cholangitis in non-neoplastic liver.

Histology is often key in determining whether a bile duct tumor is primary or secondary, as it may be difficult to differentiate based on clinical presentation or radiologically. On histology, primary cholangiocarcinomas are known to express CK7. Meanwhile, tumors with an anatomical origin in the colon will not express CK7 [11,13,14]. In this index case, the biopsies were negative for CK7 but positive for CK20 and CDX2. This created a diagnostic dilemma as the patient had no known history of a colon cancer that could correlate with this immunohistochemical finding. Furthermore, a small portion of cholangiocarcinomas can exhibit intestinal differentiation, including expression CK20 and CDX2 [14]. The distinction between primary cholangiocarcinoma of intestinal type and a colon cancer could not be achieved by morphological or immunohistochemical staining pattern only. It was therefore important to perform endoscopic evaluation to rule in or rule out the presence of an unknown colon cancer.

CRC is the second leading cause of cancer-related deaths in the United States, and therefore this index case reveals a previously unreported manifestation of a clinically relevant disease. It is important to ensure age-appropriate screening, starting at age 45 years, in average-risk adults [15]. The goal of screening is to detect cancer early or remove precancerous polyps before they become cancerous. Colonoscopy is considered the gold standard for CRC screening, but other recommended methods include stool-based tests and CT colonography [15,16]. The patient in this case did not receive appropriate screening as an outpatient, and this case also underscores that CRC is more likely to be diagnosed at a later stage in this cohort of patients.

## Conclusions

The existing literature reports exceedingly uncommon cases of CBD tumors being diagnosed after CRC, but this is the first case to report CBD tumor leading to the diagnosis of CRC. In this index case, painless obstructive jaundice was a presenting manifestation, but this is not typically associated with CRC. This case shows that in the exceedingly rare cases in which CRC metastasizes to the common bile duct, painless obstructive jaundice may occur. Clinicians should be aware of this unique presentation so that similar cases are not missed and for future cases to be further characterized and studied.
